# 2, 3, 5, 4’-tetrahydroxystilbene-2-O-beta-D-glucoside protects against neuronal cell death and traumatic brain injury-induced pathophysiology

**DOI:** 10.18632/aging.203958

**Published:** 2022-03-21

**Authors:** Yu-Hsin Chen, Yen-Chou Chen, Yu-Tang Chin, Ching-Chiung Wang, Ling-Ling Hwang, Liang-Yo Yang, Dah-Yuu Lu

**Affiliations:** 1 Graduate Institute of Medical Sciences, College of Medicine, Taipei Medical University, Taipei 11031, Taiwan; 2 Department of Physiology, School of Medicine, College of Medicine, Taipei Medical University, Taipei 11031, Taiwan; 3 School of Dentistry, College of Oral Medicine, Taipei Medical University, Taipei 11031, Taiwan; 4 School of Pharmacy, College of Pharmacy, Taipei Medical University, Taipei 11031, Taiwan; 5 Department of Physiology, School of Medicine, College of Medicine, China Medical University, Taichung 404333, Taiwan; 6 Laboratory of Neural Repair, Department of Medical Research, China Medical University Hospital, Taichung 40447, Taiwan; 7 Department of Pharmacology, School of Medicine, College of Medicine, China Medical University, Taichung 404333, Taiwan; 8 Department of Photonics and Communication Engineering, Asia University, Taichung 41354, Taiwan

**Keywords:** traumatic brain injury, Chinese herb, apoptosis, cognitive dysfunction, excitotoxicity

## Abstract

Traumatic brain injury (TBI) is a global health issue that affects at least 10 million people per year. Neuronal cell death and brain injury after TBI, including apoptosis, inflammation, and excitotoxicity, have led to detrimental effects in TBI. 2, 3, 5, 4’-tetrahydroxystilbene-2-O-beta-D-glucoside (THSG), a water-soluble compound extracted from the Chinese herb Polygonum multiflorum, has been shown to exert various biological functions. However, the effects of THSG on TBI is still poorly understood. THSG reduced L-glutamate-induced DNA fragmentation and protected glial and neuronal cell death after L-glutamate stimulation. Our results also showed that TBI caused significant behavioral deficits in the performance of beam walking, mNSS, and Morris water maze tasks in a mouse model. Importantly, daily administration of THSG (60 mg/kg/day) after TBI for 21 days attenuated the injury severity score, promoted motor coordination, and improved cognitive performance post-TBI. Moreover, administration of THSG also dramatically decreased the brain lesion volume. THSG reduced TBI-induced neuronal apoptosis in the brain cortex 24 h after TBI. Furthermore, THSG increased the number of immature neurons in the subgranular zone (SGZ) of the dentate gyrus (DG) of the hippocampus. Our results demonstrate that THSG exerts neuroprotective effects on glutamate-induced excitotoxicity and glial and neuronal cell death. The present study also demonstrated that THSG effectively protects against TBI-associated motor and cognitive impairment, at least in part, by inhibiting TBI-induced apoptosis and promoting neurogenesis.

## INTRODUCTION

Traumatic brain injury (TBI) is a serious global health problem that involves structural damage and causes changes in various functional and psychological outcomes [1]. TBI causes brain tissue damage directly and follows a variety of pathophysiologies, such as inflammation, apoptosis, oxidative stress, neuronal cell death, and excitotoxicity [2, 3]. DNA damage affects apoptosis, which is a crucial cellular link to oxidative stress following TBI [4]. Due to the difficulty of TBI prevention in clinical cases, studies focusing on post-injury and secondary injury of TBI regulation, such as DNA damage and repair are important [5]. Secondary injury in TBI management is a key point in avoiding significant impairment and understanding the pathophysiology of TBI [6].

Glutamate is the major excitatory neurotransmitter in the central nervous system (CNS), which plays a critical signal in neuronal-neuronal and neuronal-glial communication. The concentration of glutamate in the presynaptic neurons is approximately 100 mM [7], and 2 mM in the synaptic after synaptic release [8]. In physiological condition, the level of glutamate in the cerebrospinal fluid (CSF) is about 1 μM [7], but they can be elevated under pathological conditions, such as stroke, trauma, and meningitis [9]. Previous studies reported that TBI increased the concentration of glutamate in the brain, which may cause excitotoxicity and further brain damage [10, 11]. TBI has been reported to acutely trigger glutamate-induced neuronal cell death [12]. Using microdialysis, evidence shown that extracellular glutamate levels were much higher in rats with severe TBI as opposed to non-injured control rats [13]. In a clinical study, severe TBI results in elevated glutamate levels in CSF and persists for several days [14]. Glutamate may bind and activate the ionotropic N-methyl-D-aspartate (NMDA), the α-amino-3-hydroxy-5-methyl-isoxazole propionate (AMPA), and the kainate or metabotropic glutamate receptors on both neurons and astrocytes [15]. In addition, increased extracellular glutamate exacerbates the homeostasis of astrocytic ionic conductance following TBI [16]. Importantly, high glutamate concentration leads to pathological cell swelling and cell death in hippocampal cultures [17]. Moreover, the activity of the NMDA receptor signaling pathways promotes glutamate-evoked neurotoxic effects and cellular degeneration [18]. Treatment with NMDA antagonists in the acute stage ameliorated TBI and decreased neuronal apoptosis in a rodent model [19]. A recent report also indicated that controlling excitotoxicity during brain trauma is a promising strategy for improving TBI [20].

Neuronal survival is affected by the functions of astrocytes during brain injury, including maintaining homeostasis of glutamate levels [21]. In addition to neuronal cell death, glutamate also causes damage to glial cells, especially astrocytes [22]. Reactive astrocytes are considered as therapeutic targets for various neurological disorders [23]. A previous study also revealed that decreasing astrocyte activation improves neurobehavioral function [24]. Regulating autophagy in astrocytes and neurons has also been reported to improve functional recovery after TBI [25]. Importantly, reactive astrocytes have been recognized to contribute to post-traumatic tissue repair and synaptic remodeling after TBI [26]. Moreover, activation of local astrocytes serves as a compensatory response that modulates tissue damage and recovery following TBI [27]. TBI has been suggested to cause astrocyte damage which may aggravate the outcomes of patients with TBI [11]. Increasing evidence has revealed that several detrimental neuronal outcomes of TBI can modulate and improve recovery by regulating metabolic and inflammatory states [28, 29]. In addition, the administration of antioxidants has also been recognized to exert neuroprotective effects in TBI therapeutics [30, 31].

A water-soluble natural compound, 2, 3, 5, 4’-tetrahydroxystilbene-2-O-beta-D-glucoside (THSG), a low molecular weight glycosylated resveratrol, which is extracted from the traditional Chinese herb, Polygonum multiflorum (PM), has been found to exert various beneficial anti-oxidative stress and anti-inflammatory effects. [32–34]. Recently, treatment with THSG has been reported to effectively ameliorate CNS injury [35]. Moreover, THSG has been found to inhibit inflammatory responses in brain microglial cells [36] and protect hippocampal neurons against staurosporine-induced neurotoxicity [37]. Importantly, evidence generated in a mouse model, has shown that THSG inhibits neuronal apoptosis and downregulates NMDA receptor activity, thereby relieving chronic inflammatory pain [38]. In this study, we investigated whether THSG affected the viability of astrocyte cell lines and primary cortical neurons. We also evaluated the effects of the administration of THSG in a TBI mouse model by investigating the pathophysiology and behavioral outcomes.

## RESULTS

### Protective effects of THSG on glutamate-induced excitotoxicity in astrocytes and cortical neuron

**Figure 1:**
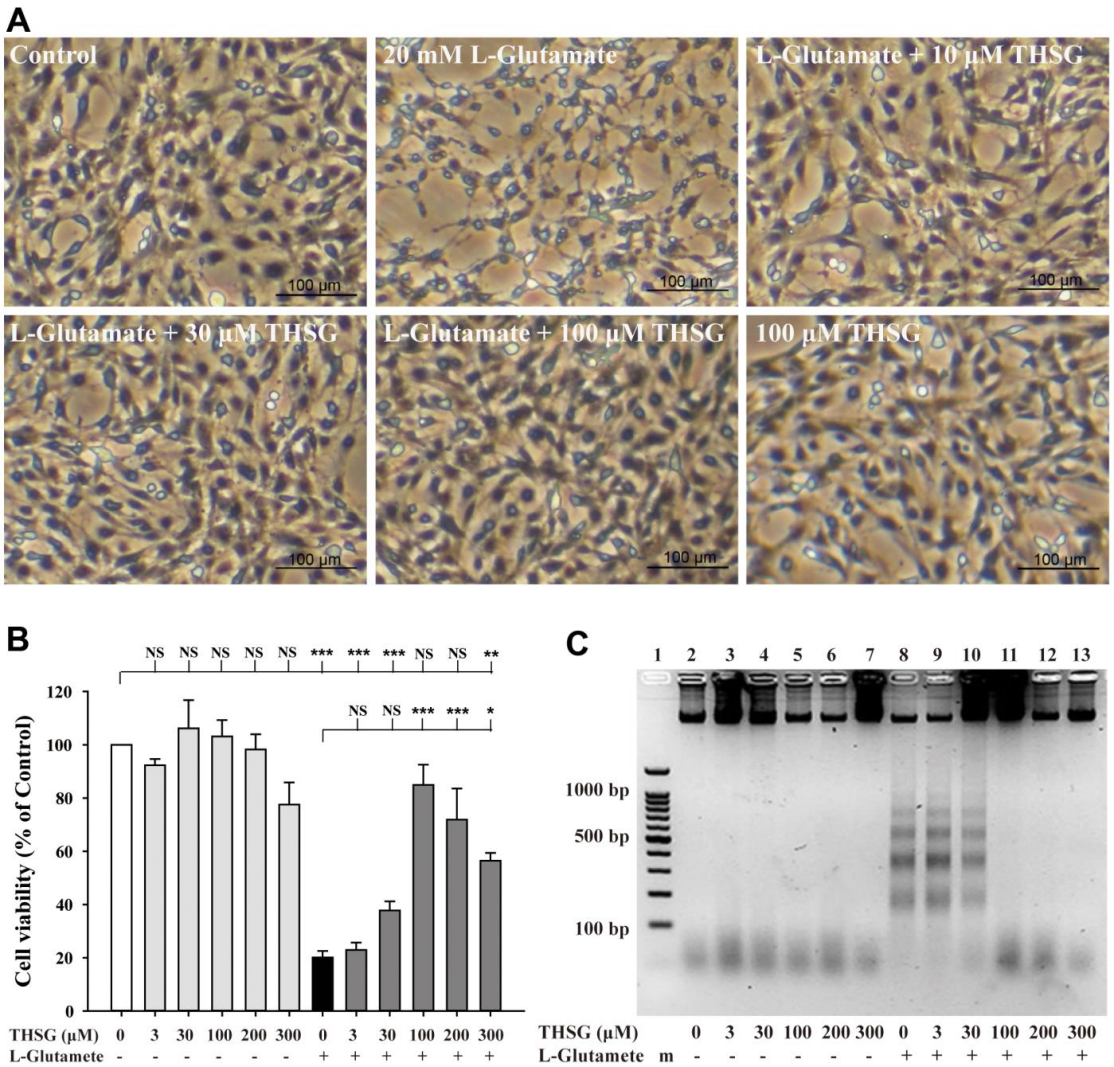
**Effects of THSG on glutamate-induced DNA fragmentation and excitotoxicity.** C6 neural glioma cells were treated with various concentrations of THSG (10, 30, or 100 μM) for 2 h and then treated with L-glutamate (20 mM) for 24 h. C6 glioma cell morphology images are shown in (**A**). Cells treated with 20 mM L-glutamate for 24 h caused drastic cell death, as demonstrated by the poor and shrunken cell morphology. Scale bar = 100 μm. Cell viability measured by the MTT assay is shown in (**B**) (n = 4). Note: THSG significantly rescued glioma neural cells from glutamate neurotoxicity, and the best effective dose of THSG was 100 μM, which completely prevented glutamate-induced cell death. NS = no significant difference; ** P > 0.01; *** P < 0.001 between the groups. Statistical analysis was performed using ANOVA for repeated measures followed by Tukey’s test of least significant difference. NS = no significantly difference; *, P > 0.05; **, P < 0.01; ***, P < 0.001. (**C**) Gel electrophoresis showing the effects of THSG at a range of concentrations (3-300 μM) on L-glutamate-induced DNA fragmentation in glioma neural cells.

**Figure 2:**
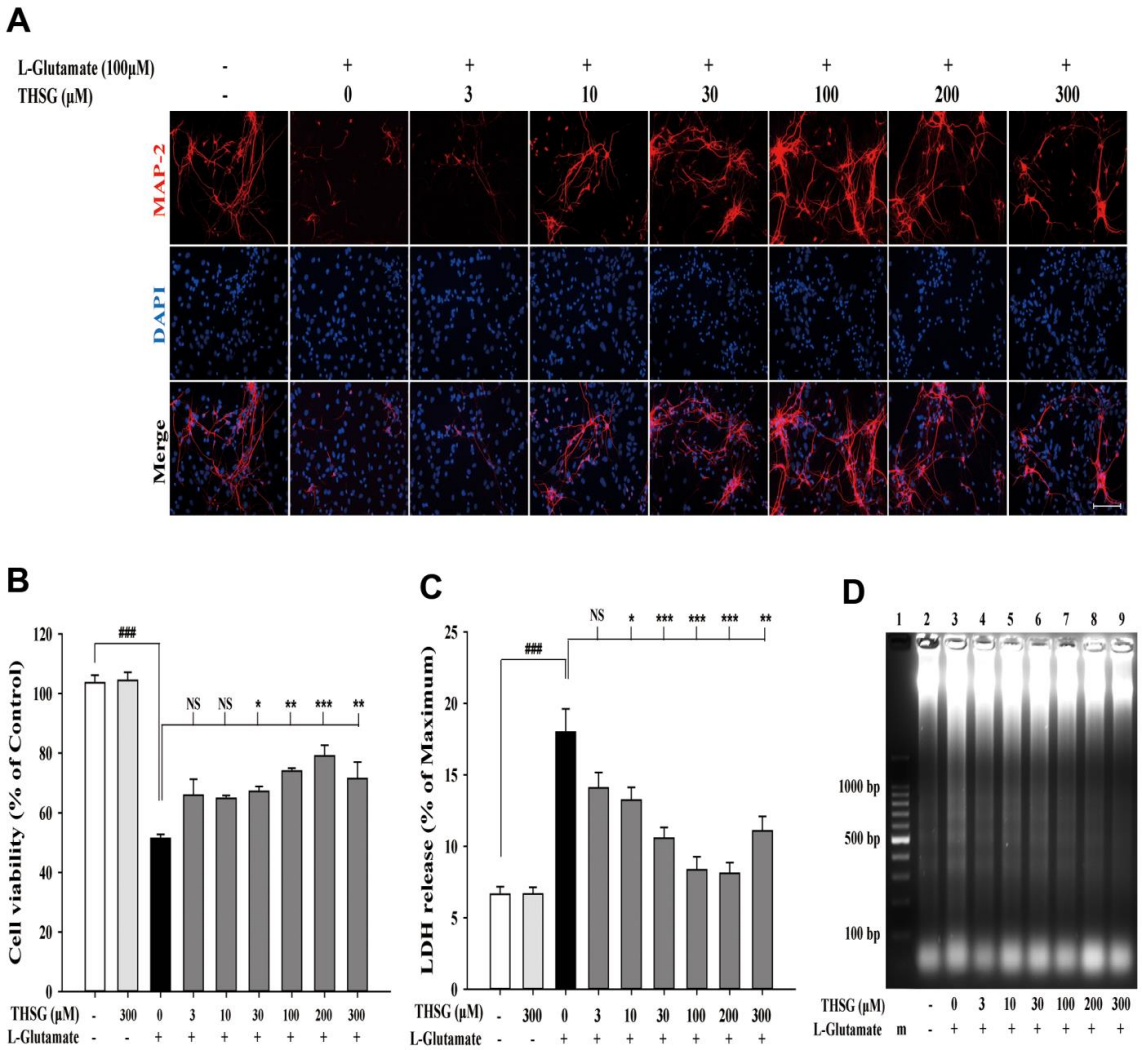
**THSG ameliorates glutamate-induced neuronal morphological change and excitotoxicity in primary cortical neurons.** (**A**) Immunofluorescence images of treatment with or without various concentrations of THSG for 2 h followed with 100 μM L-glutamate for another 24 h; Staining performed with the primary antibody anti-MAP-2 (specific marker of neuronal dendrites, red) and a nuclear-specific dye DAPI (blue). Cells treated with L-glutamate for 24 h causes dendritic shrinkage; application of THSG 2 h prior to the treatment with glutamate improves the glutamate-induced neuron shrinkage. Merged images show the labeling co-localization. Scale bar = 100 μm. Cells were pre-treated for 2 h with THSG prior to treatment with L-glutamate for another 24 h; cell viability of primary cortical culture neurons was evaluated by MTT assay (**B**) and by lactate dehydrogenase (LDH) assay (**C**) (n = 4). Statistical analysis was carried out using ANOVA for repeated measures, followed by Tukey’s test of least significant difference. NS = no significant difference; *, P > 0.05; **, P < 0.01; ***, P < 0.001. (**D**) Gel electrophoresis showing the effects of THSG at a range of concentrations (3–300 μM) on L-glutamate-induced DNA fragmentation in primary cortical culture neurons.

To investigate the effects of THSG on neuronal cells, we evaluated the survival of glioma neural cells and primary cortical neuronal cells after glutamate-induced cell death. As shown in Figure 1A, glutamate caused poor and shrunken cell morphology in glioma neural cells. Moreover, treatment with THSG effectively reversed glutamate-induced morphological alterations and excitotoxicity (Figure 1A). The morphology of cells treated with 100 μM THSG was similar to that of normal cells. In comparison, application of THSG for 2 h prior to the treatment with glutamate, effectively attenuated the glutamate-induced death of glioma neural cells, which also corresponded to dose-dependent normal cell morphology. Furthermore, stimulation with glutamate also resulted in drastic cell death when compared to cells without treatment (F[11,36] = 24.188, P < 0.001) (Figure 1B). Importantly, MTT results indicated that treatment of THSG at concentrations of 100 to 300 μM revealed a dramatic improvement in the cell survival rate in glutamate-induced cell death (P < 0.001 versus cells with glutamate treatment). In addition, there was no significant difference of THSG treatment on glutamate-induced cell death at concentration of 3 to 30 μM compared to the vehicle control group (Figure 1B). A previous study established that DNA damage following TBI contributes to programmed cell death and long-term functional deficits [39]. Thus, we conducted a DNA fragmentation assay to evaluate whether THSG protects glioma neural cells against glutamate-induced DNA fragmentation. As shown in Figure 1C, glutamate markedly induced DNA fragmentation in the glioma neural cells. Surprisingly, treatment with THSG (at concentrations of 100–300 μM) dramatically prevented glutamate-induced DNA fragmentation (Figure 1C, lanes 11, 12, and 13). In addition, no DNA fragmentation was detected after THSG treatment alone from 3 to 300 μM (Figure 1C, lanes 2–7). We also determined the effects of THSG in primary cortical neuronal cells after glutamate stimulation. As shown in the anti-MAP-2 immunostaining images, administration of L-glutamate for 24 h caused dendritic shrinkage in primary cortical neurons (Figure 2A). Conversely, treatment with THSG dramatically reduced the glutamate-induced dendritic shrinkage (Figure 2A). In particular, treatment with THSG at doses higher than 100 μM showed stronger neuronal dendritic outgrowth, similar to that of control neurons (Figure 2A). Furthermore, stimulation of L-glutamate for 24 h decreased cell viability (F[8,27] = 30.624, P < 0.001) (Figure 2B), which was effectively inhibited by THSG treatment (Figure 2B). Moreover, glutamate also increased the release of LDH from cortical neuronal cells (F[8,27] = 16.358, P < 0.001) (Figure 2C) compared to the control group. In contrast, application of THSG 2 h prior to the treatment with L-glutamate for 24 h not only improved the glutamate-induced neuron shrinkage morphology but also rescued the glutamate-induced LDH release in primary cortical culture neurons (Figure 2C). Importantly, the protective effects of THSG on primary cortical neuronal cells were more pronounced in the LDH assay, even at a lower dose of THSG (10 μM), which significantly attenuated glutamate induced LDH release (P < 0.05). Moreover, primary cortical neurons treated with 100 μM and 200 μM THSG showed the strongest effect in reducing glutamate induced LDH release (P < 0.001). This phenomenon was similar with the morphological alterations. Moreover, stimulation with L-glutamate for 24 h also induced DNA fragmentation in primary cortical neuronal cultures (Figure 2D, lane 3). Remarkably, treatment with THSG alleviated DNA fragmentation induced by glutamate (Figure 2D, lanes 4–8). These results suggest that THSG exerts beneficial effects on glutamate induced excitotoxicity, DNA cleavage, and cell death in C6 glioma cells and cortical neurons.

### THSG improves neurological function outcomes and reduces lesion volume in a TBI mouse model

**Figure 3:**
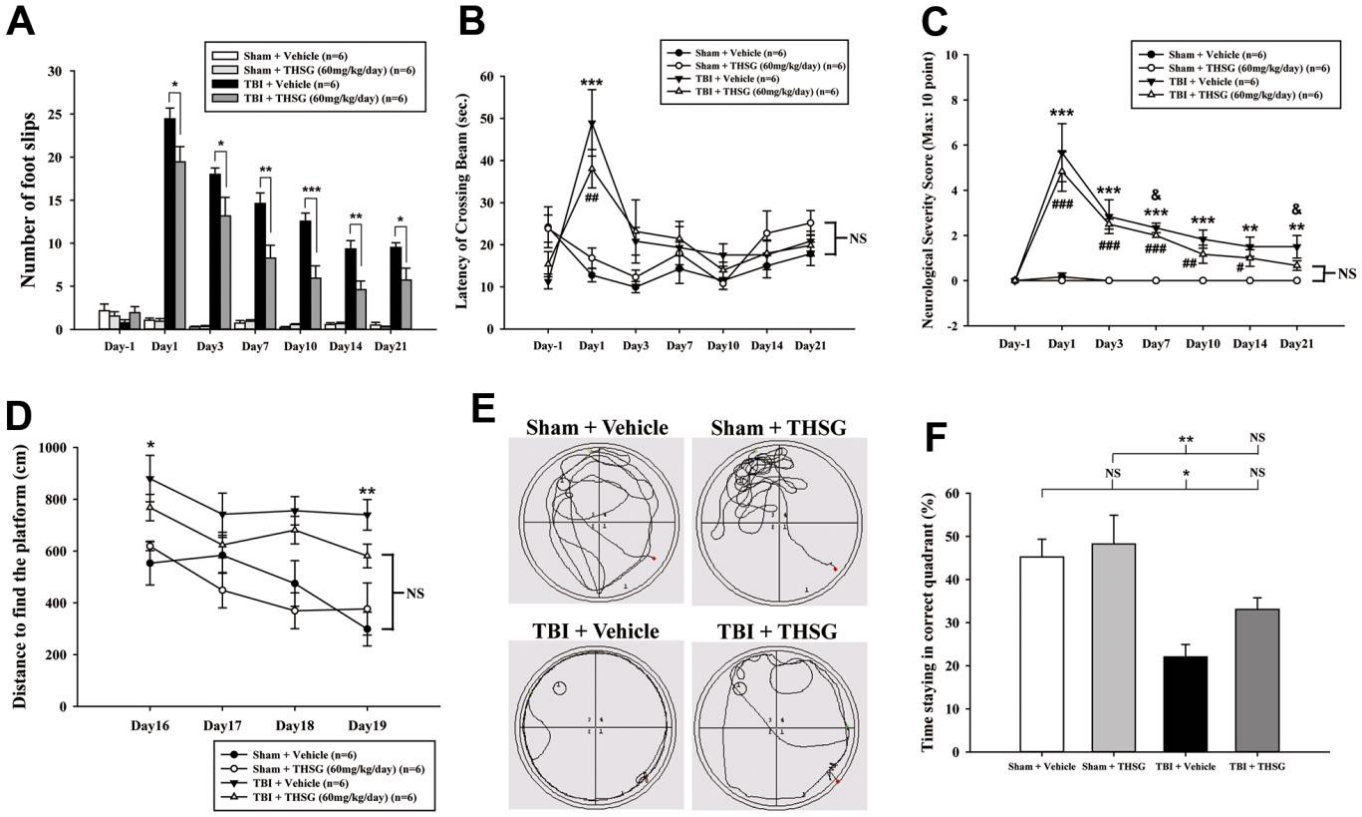
**Administration of THSG improves neurological outcomes and cognitive functions following post-TBI.** (**A**) Evaluation of motor coordination by beam-walking test in THSG treatments after TBI. Data represented as the mean ± SEM (n = 6 per group). *, P < 0.05; **, P < 0.01 and ***, P < 0.001 TBI + Vehicle vs. TBI + THSG group. (**B**) Latency of beam crossing in beam-walking task. Data represented as the mean ± SEM (n = 6 per group). NS = no significantly difference between groups; ***, P < 0.001 TBI + Vehicle vs. Sham + Vehicle group; ##, P < 0.01 TBI + THSG vs. Sham + Vehicle group. (**C**) Neurological function measured by mNSS. Data represented as the mean ± SEM (n = 6 each group). NS = no significantly difference between TBI + THSG and Sham + Vehicle group; **, P < 0.01 and ***, P < 0.001 TBI + Vehicle vs. Sham + Vehicle group. #, P < 0.05; ##, P < 0.01 and ###, P < 0.001 TBI + THSG vs. Sham + Vehicle group. &, P < 0.05 TBI + Vehicle vs. TBI + THSG group. (**D**) Cognitive performance measured by the Morris water maze test. Data represented as the mean ± SEM (n = 6 per group). NS = no significantly difference between TBI + THSG and Sham + Vehicle group; *, P < 0.05 and **, P < 0.01 vs. Sham + Vehicle group. (**E**) Representative images showed the swimming path of the maze task without platform at day 19 following THSG treatments. The circle in the specific quadrant outlines the original position of the hidden platform. Once in the probe trial, mice were released at the opposite site (red spot) for 60 seconds. (**F**) Spatial memory evaluated by probe test of Morris water maze. Data represented as the mean ± SEM (n = 6 per group). NS = no significantly difference, *, P < 0.01 vs. Sham + Vehicle group. **, P < 0.01 vs. TBI + Vehicle group.

**Figure 4:**
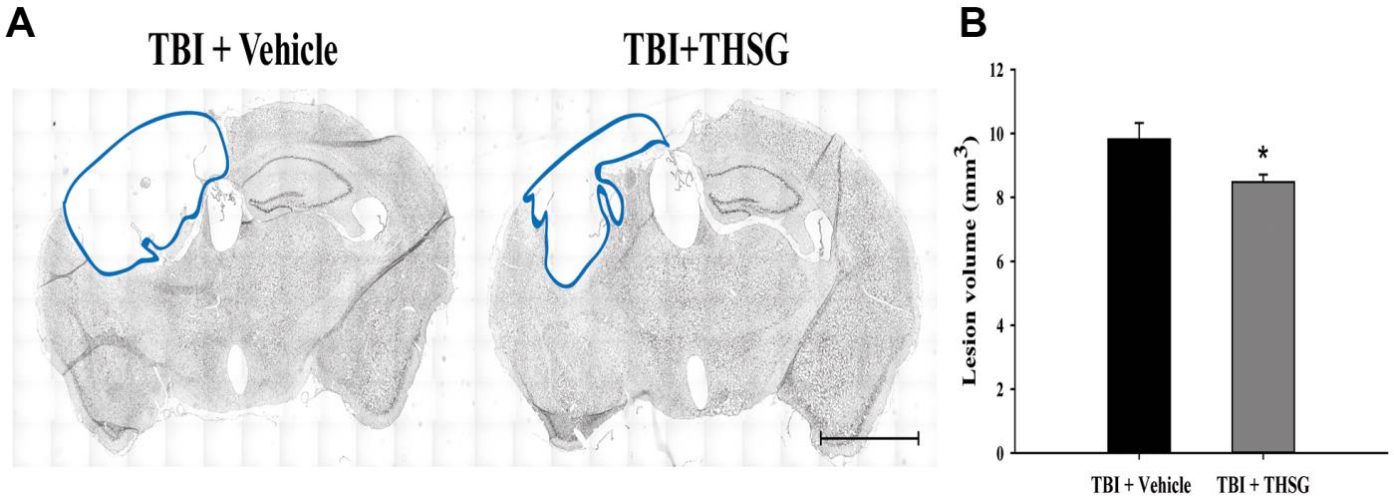
**THSG treatment reduces cortical lesion volume post TBI.** (**A**) Microphotograph representing the brain section of TBI and TBI with THSG treatment at 21 days post TBI. Lines indicate the areas of the lesion was measured (blue). Scale bar = 2 mm. (**B**) Quantification of the lesion size from sections. Data represent the mean ± SEM. *, P < 0.05 compare with TBI + Vehicle group (n = 6 for each group).

To assess the effects of THSG on TBI, we assessed cognitive and motor function using the modified neurological severity score (mNSS), beam-walking test, and Morris water maze task in a TBI mouse model. As shown in Figure 3A, mice that underwent TBI for 24 h had a significantly increased number of foot faults across the beam compared to the sham group (F[3,20] = 124.457, P < 0.001) in the beam walk test. Conversely, administration of THSG (60 mg/kg/day) post-TBI dramatically decreased the foot slips on the beam for the 21-day period (Figure 3A). Additionally, the latency of the crossing beam increased after TBI on day 1 compared with the sham vehicle group (F[3,20] = 13, P < 0.001) (Figure 3B). There was no significant difference between any group between 3 days and 21 days following TBI, indicating that the ability to walk on the beam is recovered to a point similar to that in the sham group (Figure 3B). Moreover, the neurological function measured by mNSS indicated that TBI evoked motor deficits compared with the sham vehicle group (Figure 3C). The loss of reflex reaction, balance disabilities, and reduced seeking behaviors were observed in the TBI group. Importantly, administration of THSG improved TBI-associated motor deficits, and the TBI mice treated with THSG showed better performance at 21 days, similar to the performance in the sham vehicle groups (F[3,20] = 6.887, P > 0.05) (Figure 3C). In the cognitive task, the distance that the mice swam to find the hidden platform was decreased in the vehicle groups compared with that in the TBI mice (Figure 3D). Furthermore, administration of THSG mildly reduced the swimming distance following TBI, and TBI mice treated with THSG showed no significant difference at day 19 compared with the sham vehicle group (P = 0.076). However, the TBI group showed poor outcomes in the special memory test compared with the sham vehicle group (F[3,20] = 7.988, P < 0.01) (Figure 3D). Furthermore, the probe test was evaluated once for 1 minute with platform removal after the final acquisition trial. The TBI mice showed disability and seeking behavior to find the platform (Figure 3E), which remarkably reduced the percentage of swimming time in the correct quadrant (Figure 3F; F[3,20] = 7.557, P < 0.05). Importantly, THSG treatment after TBI improved the swimming path of the water maze task without the platform (Figure 3E). The mice tended to increase the time spent in the correct quadrant compared to the TBI with THSG group (Figure 3F). Following behavioral tests, we further explored whether THSG ameliorates brain lesions in TBI animals. As shown in Figure 4A, animals with TBI for 21 days showed a large lesion area and lost most of the hippocampus in the ipsilateral region by Nissl staining. Moreover, the quantitative results showed that administration of THSG significantly reduced the brain lesion volume following TBI (t = 2.251, P < 0.05) (Figure 4B). These data demonstrate that THSG treatment exerts beneficial effects on motor and cognitive performance following TBI. In addition, this study also showed that THSG effectively improved TBI-evoked brain lesions.

### THSG treatment reduces TBI-induced neural apoptosis after TBI

**Figure 5:**
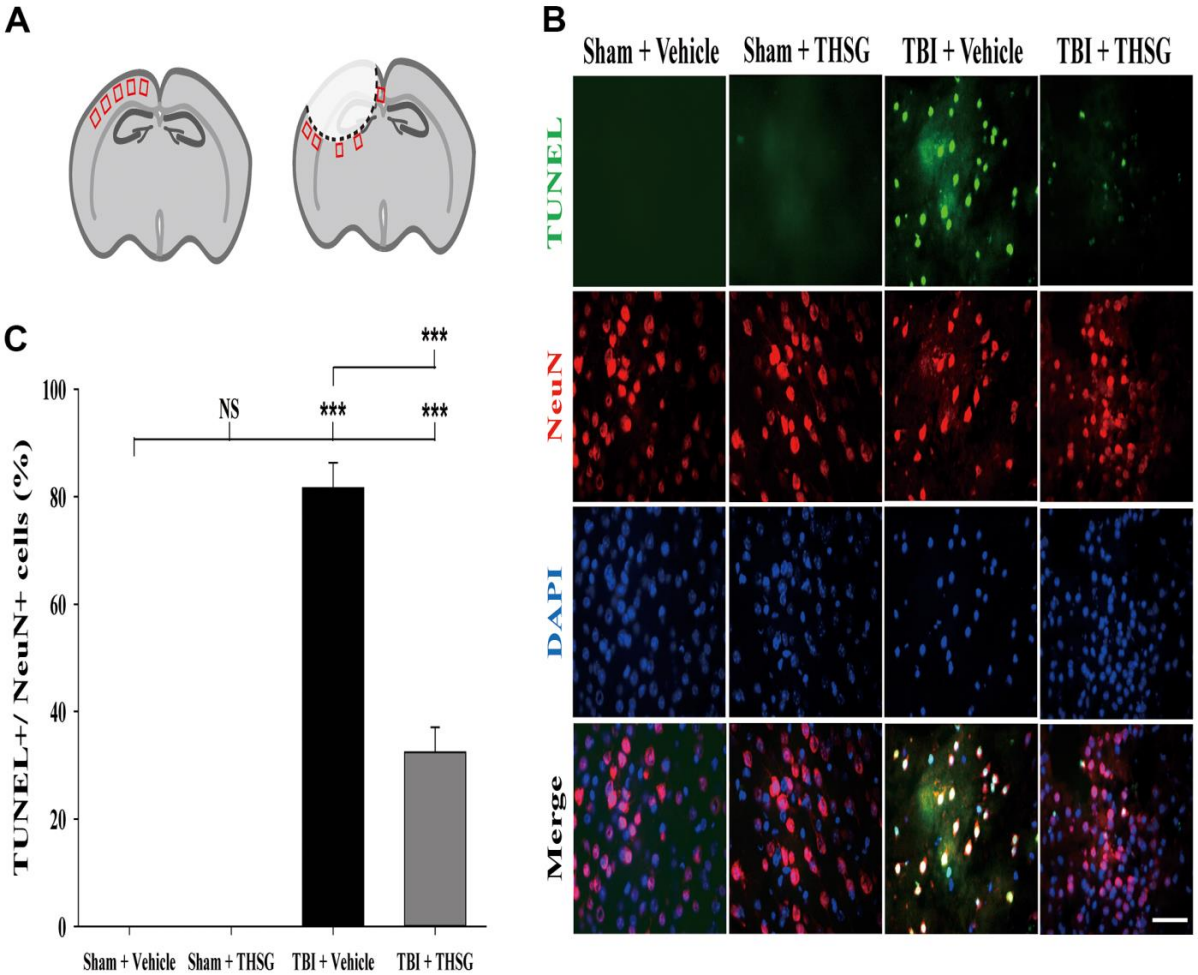
**Administration of THSG decreased TBI-induced neural apoptosis in the brain cortex post TBI.** (**A**) Schematic illustration of regions of interest (ROIs) in cerebral cortex with sham (left) or TBI (right). The sampling areas are shown in red squares. (**B**) Representative double staining immunofluorescence with TUNEL assay (green) and NeuN (a maker for neurons, red) and DAPI (blue) counterstain in brain cortex from the Sham + vehicle, Sham + THSG, TBI + vehicle, and TBI + THSG group. Labeling co-localization is shown as yellow in the merged images. Scale bar = 50 μm. (**C**) Quantification of the co-localization of the TUNEL positive and NeuN, as well as the counterstaining with DAPI in cortical brain tissues from the Sham + vehicle, TBI + vehicle, and TBI + THSG groups. Data are expressed as the mean ± SEM (n = 6 per group). ***, P < 0.001 between groups.

A growing body of evidence suggests that neuronal apoptosis aggravates TBI damage in the pathological processes, which can be promoted by apoptosis pathway modulation [40, 41]. We investigated whether treatment with THSG attenuated TBI-induced neural apoptosis. Mice were administered THSG (60 mg/kg) 1h following TBI, and the neural apoptotic cells were determined by double immunofluorescence staining TUNEL and a neuronal nuclear protein marker NeuN in the brain cortex and around injury site (Figure 5A), 24 h after TBI (Figure 5B). Our results showed that TBI increased the number of TUNEL-positive cells that were co-localized with neuron-specific protein counterstaining with DAPI (4',6-diamidino-2-phenylindole, a blue fluorescence nuclear staining dye) compared to the sham vehicle group (F[3,20] = 135.566, P < 0.001). Surprisingly, administration of THSG dramatically negated TBI-induced neuronal apoptosis (Figure 5C). TUNEL+/NeuN+ cells were strongly reduced after THSG treatment post TBI.

### THSG treatment increases the number of immature neurons in hippocampus after TBI

**Figure 6:**
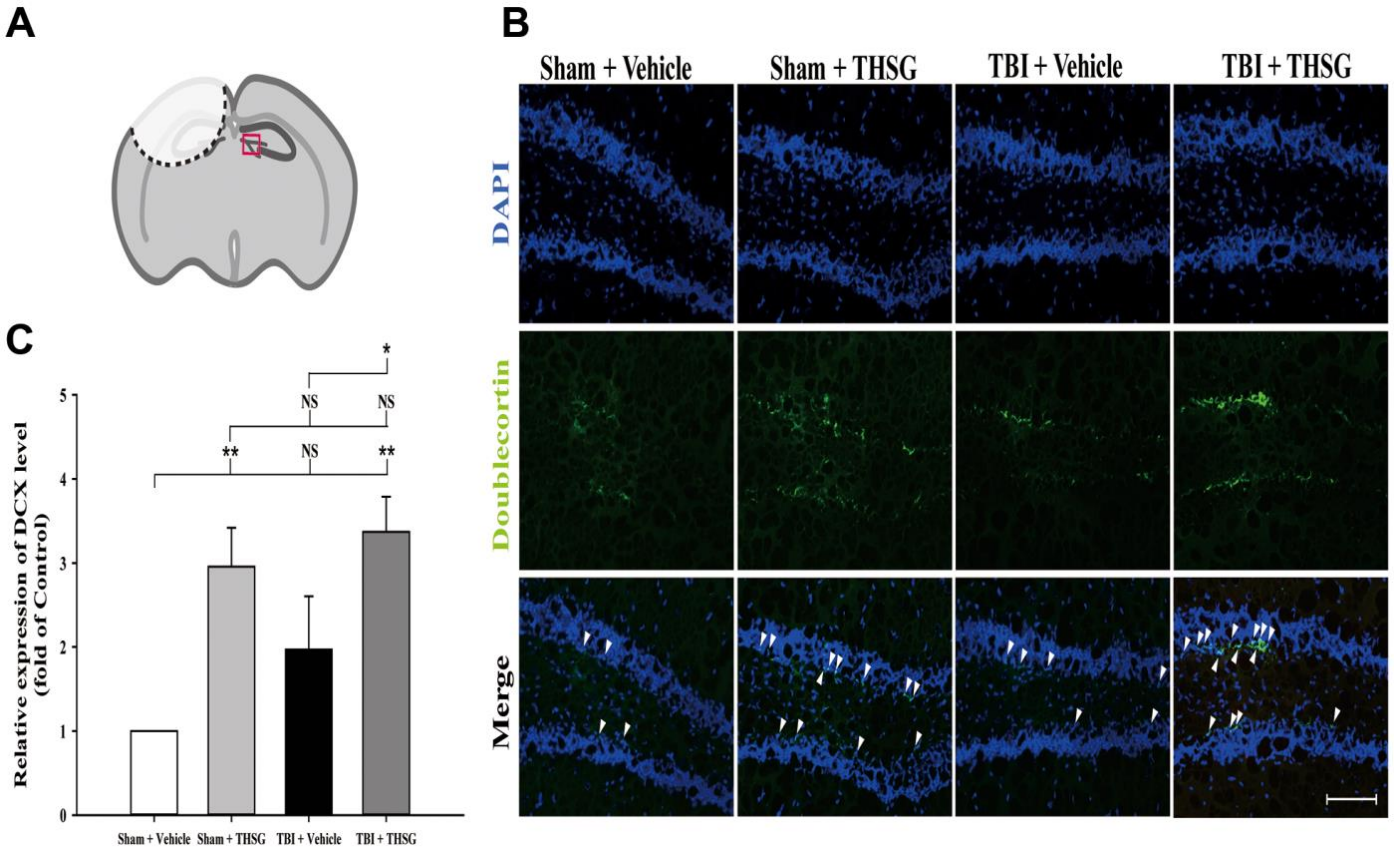
**Administration of THSG increases the number of hippocampal immature neurons in the subgranular zone of dentate gyrus post TBI.** (**A**) Illustrations of the region of interest with red square in the brain section sample. (**B**) Representative immunofluorescence staining by anti-doublecortin (DCX, a marker for immature neurons, green) and counterstained with DAPI (blue), 21 days after THSG treatments in the subgranular zone of dentate gyrus. Labeling co-localization is shown with white arrows in the merged images. Scale bar = 100 μm. Mice were divided into three groups: Sham + Vehicle, Sham + THSG, TBI + vehicle, and TBI + THSG groups. (**C**) Quantitative results of the expression of DCX shown as the fold of the Sham + Vehicle group. Data represented as the mean ± SEM (n=6 for each group). NS = no significantly difference; *, P < 0.05; **, P < 0.01 between groups.

The microtubule-associated protein doublecortin, expressed in neuroblast and immature neurons, is correlated with adult neuroplasticity and neurogenesis [42, 43]. We analyzed the expression of doublecortin, a microtubule-associated protein expressed by neuronal precursor cells and immature neurons, in the dentate gyrus of the hippocampus (Figure 6A), 21 days after TBI. As shown in Figure 6B, administration of THSG significantly increased the number of immature neurons in the subgranular zone of the contralateral hippocampus (F[3,20] = 5.695, P < 0.01) compared with the sham vehicle group. Most importantly, administration of THSG in the post-TBI group further enhanced the protein expression of doublecortin in the TBI vehicle (P < 0.05) and sham vehicle group (P < 0.01), respectively (Figure 6C). These results suggest that the administration of THSG has beneficial effects on neuronal plasticity after TBI.

## DISCUSSION

Previously, it has been reported that motor deficits, cognitive impairments, structural damage and neurodegeneration are the main symptoms in the clinical diagnosis of TBI patients, which are relevant to the control cortical impact (CCI) mouse model [44, 45]. The CCI model of TBI, which we used in this study, is frequently used in moderate to severe TBI because of the necessity of a craniotomy [46]. However, it is difficult to define the severity of experimental TBI due to the lack of appropriate parameters, and this model results in damage to multiple brain functions [47]. Morris water maze performance is a well-established method to assess pre-clinical cognitive performance and damage to neural functional networks in experimental TBI animals [48]. A previous study also reported that TBI impairs special learning [49] and cognitive deficits in a CCI mouse model [50]. Importantly, clinical study has suggested that TBI at a young age is highly correlated with later dementia acquisition [51]. Our current study supports previous findings that TBI impaired learning memory outcomes which was sustained for 21 days. Chronic administration of THSG has been reported to promote hippocampal memory and synaptic plasticity in normal mouse [52]. Interestingly, a recent study has also revealed that spatial learning and memory impairment caused by infrasound in a mouse model could be attenuated by THSG intake [35]. Furthermore, THSG has been found to ameliorate memory function and inhibit alpha-synuclein aggregation in the hippocampus of aged mice [53]. Our results further demonstrated that oral administration of THSG effectively improved the impairment of learning and memory caused by TBI.

This study also showed that post-injury intake of THSG eased the deficits in mNSS evaluation similar to healthy animals, 21 days following TBI. Remarkably, beam walking has been reported to be a more sensitive tool for motor coordination measurement than Rota-rod in some cases [54, 55]. The present study also showed that TBI impaired motor coordination in the beam walking test, but treatment with THSG recovered the impairment of motor function. Furthermore, we also used the Rota-rod test, the most common motor function task, to test the improvement of THSG administration in TBI mice. However, we did not observe a significant difference between the TBI mice treated with THSG and the vehicle control (Supplementary Figure 1). Our findings suggest that careful choice of tasks as well as parameters for evaluating motor function in severe TBI models is important in future studies. Since balancing skills are still abnormal and lack the strength of the contralateral limb in TBI animals to slips, the animal could walk across the beam without pause. This study supports the idea that administration of THSG attenuated brain damage after TBI resulting in reduced brain lesion size, a hallmark of TBI severity that could be used to predict mortality and functional outcome recovery. These results may be attributed to the pain relief ability of THSG, which may result in the ability of the mice to walk over the beam in a shorter period after treatment; however, the pain parameters were not considered in the current study. Furthermore, the smaller brain lesion volume highlights the benefits of THSG.

Adult hippocampal neurogenesis plays a crucial role in various brain functions, such as learning, memory, and cognitive function [56]. However, it has been reported that neurogenesis mostly occurs following severe TBI in the adult mouse hippocampus [57]. The microtubule-associated protein doublecortin, expressed in neuronal precursor cells and adult cortical structures, is correlated with adult neuroplasticity and neurogenesis [42, 43]. Studies have demonstrated that DCX expression in immature neurons in the contralateral hippocampal dentate gyrus is linked to functional repair and neuroplasticity in TBI [58]. Another study showed that doublecortin could be a key regulator of cerebral cortical neuronal migration and axon outgrowth [59]. Importantly, upregulation of DCX has been found to result in better clinical neurological outcomes in children with severe TBI [60]. A previous study showed that treatment with THSG protected hippocampal neuronal damage by activating the neurotrophic axis of BDNF and TrkB [61]. Moreover, THSG treatment has been reported to promote neurotrophic factor release in astrocytes [62] and upregulate glutamate transporter 1 expression in astrocytes [63]. Importantly, THSG has also been found to increase astrocyte proliferation and neurogenesis in DCX-expressing cells in the hippocampus [64]. Our results showed that THSG protected glutamate from excitotoxicity and cell death in neurons and astrocytes. We also found that oral administration of THSG increased the DCX positive neuronal precursor cells in the subgranular zone of hippocampus in both TBI and normal animals. To the best of our knowledge, this is the first study elucidating the neuroprotective effects of THSG through increasing neurogenesis in a pre-clinical TBI model.

Several studies have reported that modulation of apoptosis in a TBI model alleviates the loss of brain tissue and improves pathophysiological outcomes [65–68]. Importantly, DNA damage has recently been considered as a pathologic marker, which predicts further neurodegeneration, behind mild TBI-induced injury in the brains of rodents and human patients [69]. A previous study suggested that the accumulation of DNA fragmentation reached a peak 1 d after TBI, which further triggered apoptosis and affected DNA repair that contributes to brain tissue damage [70, 71]. Another study showed that the reduction of cellular apoptosis after TBI, which was analyzed with TUNEL assays using dexmedetomidine, maintained the neurological function in the acute phase of TBI [72]. A previous study indicated that p53-activated apoptosis following TBI causes brain cortex destruction and results in motor deficits [73]. Another study found that inhibition of neuronal apoptosis by a p53 inactivator ameliorated motor and cognitive functional deficits after TBI [74]. THSG prevents the pathogenesis of Parkinson's disease by inhibiting apoptosis and alpha-synuclein aggregation [75]. Furthermore, it has also been reported that THSG inhibited glutamate-induced apoptotic cell death in hippocampal cells [76]. Our results showed that THSG effectively rescued glutamate-induced neurotoxicity in both glioma neuronal cells and primary cortical neuronal cells. In particular, THSG can prevent DNA fragmentation from glutamate-induced neural cell lines and primary neuronal cells. Moreover, our results also revealed that THSG treatment protected neural cells from apoptosis as well as lactate dehydrogenase release. Administration of THSG in the TBI animal model is also consistent with our finding that THSG markedly inhibited DNA break and TUNEL expression 24 h after brain injury. Our present study demonstrated that THSG attenuates the detrimental deficits caused by TBI by downregulating DNA damage, neuronal apoptosis, and glutamate-induced excitotoxicity.

The application of THSG has been shown to be due to acute absorption and rapid distribution, and THSG can be detected in the brain following intravenous and oral administration [77]. THSG administered intragastrically modulated amyloid precursor protein processing in APP/PS1 transgenic mice [78]. Intragastric administration of THSG has also been reported to exert anti-amyloidogenic and neurotrophic effects in a rat model of chronic neurodegenerative brain disorder [79]. Subcutaneous injection of THSG has been found to reverse stress-induced depression in a chronic restraint stress mouse model [64]. Recently, subcutaneous injection of THSG also showed attenuated stress-induced depression in a mouse model by ameliorating the neurotrophin pathway [80]. Orally administered THSG has been reported to have protective effects against cerebral ischemia/reperfusion injury in rats [81]. Our findings support previous reports that oral administration of THSG possesses long-lasting protective capacity in the central nervous system, even though it improves cognitive outcomes after brain injury. The present study investigated the most likely properties and safe dosage of orally administered THSG on control cortical impact (CCI)-induced TBI outcomes, using motor and cognitive tests as a pilot study. Here, we suggest oral administration of 60 mg/kg THSG once daily for 21 days following post-TBI in subsequent research and clinical applications.

There were some limitations in the study. The counts of apoptotic cells may depend on different animal ages, models and timelines of study. Previous studies reported that there were 3 to 5% neuronal apoptotic events in control/sham groups which using the similar TUNEL+NeuN setup with our study [82, 83]. We have counted around 300 to 420 neuronal cells (NeuN+DAPI+) in each sample with five ROIs in both sham+vehicle and sham+THSG groups, but we didn’t observe neuronal apoptotic events (Figure 5). Our results support the previous studies that TBI induced a significant increase in TUNEL-positive neuronal cells (more than 60%) while no TUNEL-positive nuclei were observed in the sham group [84]. Importantly, there were around 70% of TUNEL-positive cells were found in severe TBI patients and none of apoptotic neurons by TUNEL staining of cerebral cortex from five control cases [85]. Further detailed study will be required to identify the actual numbers of the apoptotic neurons in CNS in the physiological condition and after TBI.

## CONCLUSIONS

The current study verified that THSG effectively protects against glutamate-induced excitotoxicity and cell death in astrocytes and cortical neurons. Our results also showed that THSG exerted neuroprotective effects against DNA damage induced by glutamate. Moreover, we identified the protective role of THSG in a TBI rodent model. To the best of our knowledge, this is the first study demonstrating that post-injury treatment with THSG improves TBI-induced behavioral impairment and brain lesion volume, which is involved in post-TBI anti-apoptosis. Our results also provide a new therapeutic strategy using THSG, which enhances neuronal plasticity following TBI. Our findings suggest that THSG may be an effective candidate against TBI-induced brain lesions leading to the improvement of motor and cognitive impairment.

## MATERIALS AND METHODS

### Animals

Adult male C57BL/6 mice (7 weeks old, 20–25 g) and pregnant female C57BL/6 mice were purchased from the National Laboratory Animal Center, Taipei, Taiwan, or from BioLASCO Taiwan Co., Ltd. All animals were housed under a constant 12 h light/dark cycle at room temperature (21–25° C) and humidity levels of 45%–50%. Food and water were provided *ad libitum*. Experiments were started after a one-week habituation period. The animal experimental protocols were approved by the Institutional Animal Care and Use Committee (IACUC) of the China Medical University (CMUIACUC-2018-086 and CMUIACUC-2020-265). All animal procedures were performed in compliance with the National Institutes of Health Guidelines for the Care and Use of Laboratory Animals.

### Drug administration

The 2, 3, 5, 4’-tetrahydroxystilbene-2-O-beta-D-glucoside (THSG) was kindly provided by Dr. Ching-Chiung Wang in the Graduate Institute of Pharmacognosy, College of Pharmacy, Taipei Medical University, Taipei, Taiwan. The extraction and purification of THSG from Polygonum Multiflorum Thunb was performed as previously described [86]. In cell culture, THSG was dissolved in DMSO directly and then treated with medium for 2 h before L-glutamate treatment. In the animal model, THSG was dissolved in deionized distilled water for animal feeding. Animals were given vehicle or THSG in 1h following TBI and sacrificed 24 h later. Another group of experimental animals continued with oral gavage once daily with vehicle or THSG (60 mg/kg/day) from the next day after TBI or vehicle until 21 days to evaluate behavioral outcomes and tissue histology. Animals were randomly divided into four groups: sham + vehicle, sham + THSG, TBI + vehicle, and TBI with THSG treatment for analyzing acute response at 24 h or evaluating the behavioral test for 21 days after brain injury.

### Animal model of TBI

Traumatic brain injury was induced by a controlled cortical impactor (CCI) TBI 0310 (Precision Systems and Instrumentation, LLC, Fairfax, VA, USA). TBI induction was described in our previous study [11]. Briefly, mice were anesthetized by intraperitoneal injection of Zoletil 50 (tiletamine hydrochloride and zolazepam hydrochloride, 25 mg/ml/kg, VIRBAC Laboratories, Carros, France) and Ilium Xylazil-100 (Xylazine, 10 mg/ml/kg, Troy Laboratories, Australia) before surgery. After shaving and disinfection, the mouse skull underwent craniotomy at the left cortex using a drill. A 3 mm diameter impact head was settled 0.5 mm posterior to the bregma and 0.5 mm lateral to the midline. The weight drops a head on the flat exposed brain surface with 2 mm impact depth, 5 m/s impact velocity, and 500 ms dwell time. After cortical brain injury, the mouse skull was replaced, and the skin was enclosed with wound clips. The animals were returned to their home cage under a heat lamp until recovery from anesthesia.

### Beam walking test

Beam walking was performed and modified as described previously [87]. An 80 cm beam was placed 50 cm above the table. Light was placed at the starting point and a black box was placed at the end of the beam to motivate the mice. The animal cage was moved to the experimental area at least 1 h before any behavioral test to allow habituation. Mice underwent two days of pre-training using a 28 mm wooden stick of four times/day, followed by a 12 mm beam 4 times/day, before the first test. In the experimental trial, the time across the 6 mm beam starting from 0 cm to 80 cm was recorded, and the number of foot slips were counted on day -1, 1, 3, 7, 10, 14, and 21 following TBI. The time spent on the beam while animals froze and/or paused for excretion was excluded. The time taken for mice to cross the stick over 1 min counted as a maximum of 60 s. After the mice reached the box at the end of the beam, they were returned to their home cages. Data were averaged of three trials on each test day.

### Modified neural severity score (mNSS)

To evaluate overall neurological deficits, the mNSS was assessed 1 d prior to and on day 1, 3, 7, 10, 14, and 21 after sham or brain injury. We used 10-points tasks of mNSS that were related to motor, reflex, sensory, balance, and seeking behavior post-injury according to a previous report [88, 89]. Each task was scored as 0 for a normal result or 1 when the animal failed to show functional impairment. The total score of each animal was recorded from 0 (intact) to 10 (maximum deficit).

### Morris water maze

The Morris water maze was used to determine learning and memory behavior, as previously described [48]. A 135 cm diameter circle tank was filled with non-fat milk at room temperature (21–24° C). Animals were treated with 60 sec/trial, four trials per day with a minimum of five-minute intervals, from random quadrants in a water tank. Visual cues were placed around the tank consistently during the tasks. Mice were gently guided to the platform where they stayed for over 30 s if they failed to find the target. After every trial was completed, the mice were immediately dried and cleaned with a towel and then put back in their home cages with warm light. Swimming path and distance covered to find the platform were measured for all animals on four consecutive days, from 16 to 19 days post-TBI. The probe test was conducted once for 60 s without a platform and with visual cues 1 h after the final hidden platform trial. The percentage of travel time in the correct quadrant was recorded.

### Rota-rod test

We used a Rota-rod device (Treadmill, SINGA Technology Corporation, Taiwan) to test the motor functions of the animals according to the description provided with the device [90]. We handled and pre-trained the mice on the rod for 15 min/day for three consecutive days before the first trial. In the test, each mouse was subjected to an accelerating speed of 300 s from 0 rpm to 50 rpm five times at one-minute intervals. The time spent on the rod one day before TBI and at 1, 3, 7, 10, 14, and 21 days after TBI was recorded. Data were averaged and represented on each experimental day.

### Perfusion and sectioning

Mice were anesthetized and then transcardially perfused with cold heparinized saline followed by 4% paraformaldehyde 24 h or 21 days post TBI. The perfused brains were rapidly removed and fixed in 4% paraformaldehyde overnight. Brain tissues were transferred to 20% sucrose until they sank and then frozen in OCT gel for sectioning. Each brain was cut into 10 μm coronal sections on a cryostat (Shandon, Thermo Fisher Scientific), and the slides were stored at -80° C for histological analysis.

### Nissl staining

To evaluate tissue loss, we measured the volume of TBI-induced neuronal loss from the injury sites using thionin staining. Selected regions of interest in six coronal sections from bregma -0.5, -1, -1.5, -2, -2.5, and -3.0 mm with a 480 μm interval were collected to detect Nissl substance. Slides were stained by rinsing with distilled water several times, transferring to 70%, 95%, 100% ethanol, and defatted with xylene. After alcohol rehydration, slides were rinsed with thionin buffer, followed by 95% ethanol with galactic acid, 95% ethanol until the color disappeared (for differentiating variables) and then in 100% ethanol for dehydration. Xylene was used, and the slides were covered with mounting solution and covered with a coverslip for tissue analysis. Photographs were taken using a Carl Zeiss microscope (Axiovert 200M, Carl Zeiss), and the volume of tissue loss area in the TBI-induced injury hemispheres was measured using Axiovision software (Carl Zeiss). The volume of tissue loss in each bregma level between the two sections was calculated using the formula: d*(A1+A2)/2 formula, where d indicates the distance between sections, and A1 and A2 are the measured areas in the two different sections. The total lesion size in the different treatments was averaged.

### Cell line and primary cortical neuronal culture

C6 glioma neural cells were purchased from the American Type Culture Collection (ATCC) (10801 University Boulevard, Manassas, VA, USA). C6 glioma cells were cultured in Dulbecco's modified Eagle's medium (DMEM) containing 10% heat-inactivated fetal bovine serum (FBS) (Gibco BRL, Grand Island, NY, USA), penicillin G (100 units/ml), and streptomycin (100 units/ml) in a humidified incubator maintained at 37° C with a continuous supply of 5% CO_2_. The medium was replaced every two days. The following test was started after the cells reached 100% confluence.

Primary cortical neuronal cell culture was performed using a previously described method with some modifications (Brewer et al., 1993). Time-pregnant C57BL/6 (E15-16) were anesthetized and sacrificed by cervical dislocation. The cerebral cortex from the embryonic fetus was carefully dissected and transferred to the medium on an ice-cold stage. Tissues were gently minced using sterile surgical micro scissors, digested in 0.25% trypsin, and pipetted for dissociation of cells. After centrifugation, cells were re-suspended in HG-DMEM containing 100 U/mL penicillin-streptomycin solution and 10% fetal bovine serum at an appropriate density of cells and grown on poly l-Lysine-coated plates in a 5% CO_2_ humidified incubator at 37° C. HG-DMEM was changed to neurobasal medium containing 2% B27 supplement with antibiotics after four hours of incubation. Cells were changed to half of the medium at 4 days *in vitro* (DIV) and the entire medium was changed at DIV7 during incubation. Cortical neurons were maintained in primary culture for 7–8 days for use in the experiments.

### Cell viability assay

MTT (3-(4,5-dimethylthiazol-2-yl)-2,5-diphenyl tetrazolium bromide) assay was used to detect mitochondrial reductase to determine the cell metabolic activity, as previously described [91, 92]. C6 glioma cells were seeded at a density of 6×10^3^ cells/well in 96-well plates for 24 h. After pre-treatment for 2 h with dose-dependent THSG or vehicle, 20 mM L-glutamate or vehicle were then added for a further 24 h. Cells were washed twice with phosphate-buffered saline (PBS); MTT solution (M2128, Sigma) was then added to the medium for an additional 4 h at 37° C. The supernatant was removed, and the dark blue formazan crystals were dissolved with 0.1 N HCl in isopropanol at room temperature. The absorbance was read at 570 nm using a microplate reader. Similarly, 6×10^4^ embryonic primary cortical neurons/well were seeded in 96-well plates for 7 days and then treated with vehicle or THSG 2 h prior to another 24 h treatment with 100 μM L-glutamate (G5889, Sigma). MTT procedures were performed according to the manufacturer’s instructions (M6494, Life Technologies), and each well was quantified for analysis at a wavelength of 540 nm.

### DNA fragmentation

DNA fragmentation tests were used to determine DNA integrity as a sign of cell death, based on published reports [93]. C6 glioma cells were seeded at 6×10^4^ cells/ml in 6-well plates and incubated for 24 h before the experiment. For primary cortical neurons, 4×10^6^ cells/ml were cultured for treatment until DIV7. Cells under the different treatments were collected and washed with cold PBS, then lysed in 100 μl of lysis buffer (100 mM NaCl, 50 mM Tris-HCl pH8.0, 0.5 M EDTA pH8.0, and 0.5% SDS) with proteinase K (P2308, Sigma) for 3 h at 56° C, and then treated with 0.5 mg/mL RNase A for an additional 1 h at 56° C. DNA was extracted using phenol/chloroform/isoamyl alcohol (25:24:1, v/v, Sigma). Equal amounts of DNA and loading buffer were loaded onto a 1.8-2% agarose gel containing SYBR Safe (1:10000, Invitrogen) and run at 100 V for 40 min in TAE buffer; the gel was then observed and photographed under UV light.

### LDH cytotoxicity assay

Cytotoxicity was assessed based on the release of lactate dehydrogenase (LDH) using a cytotoxicity detection kit (ab65393 LDH Cytotoxicity Assay Kit, Abcam, USA), a colorimetric assay for the quantification of cell death based on the measurement of lactate dehydrogenase activity released from the cytosol of damaged cells into the supernatant. Primary neurons were seeded at a density of 6×10^4^ cells per well in 96 well plate for 7 days. After experimental treatment, each well was mixed with LDH reaction buffer and gentle shaking at room temperature for 30 min. Cells without any treatment served as low controls. A high control was treated with the cell lysis solution. Each sample was assayed in triplicate, and the wavelength was measured at 450 nm. To determine the percentage of cytotoxicity, the average absorbance values of the triplicate readings were calculated, and the values were substituted into the following equation: Cytotoxicity (%) = (test sample value – low control value / high control value – low control value) × 100.

### TUNEL assay for neurons

Terminal deoxynucleotidyl transferase (TdT) dUTP nick end labeling (TUNEL) was used to detect apoptotic cells by labeling the 3’-hydroxyl terminus of DNA strand breaks. To test the anti-apoptotic effects of THSG on neurons, mouse brain tissue was collected for cryosection 24 h after TBI. Slides of mouse brains were warmed, washed with PBS, and permeabilized with 0.1% Triton-X 100 and 0.1% (w/v) sodium citrate for 20 min. Slides were then co-stained with neuron markers and detected as immunofluorescence following the protocol of the *in situ* Cell Death Detection Kit (Roche Diagnostics GmbH, Mannheim, Germany). Briefly, sections were blocked with 1% normal goat serum in PBS containing 0.1 % Tween 20 for 40 min at room temperature. The sections were then added with the primary anti-mouse monoclonal antibody, NeuN (1:1000, ab104224, Abcam) and incubated overnight at 4° C. After rinsing with PBS, the sections were incubated with the secondary antibody, conjugated anti-mouse IgG (H+L) (1:500 A21424, Alexa Fluor 555 Conjugate) for 1 h at room temperature in the dark. The sections were rinsed and the mixture of label and enzyme solution was added for 90 min in a dark 37° C humidity incubator. Sections were rinsed and mounted with Vectashield Antifade Mounting Medium with DAPI (H-1800, Vector Laboratories, Burlingame, CA, USA) to analyze the number of fluorescence-expressing cells from five ROI images of each brain section.

### Immunofluorescence

We evaluated protein expression levels 21 days after TBI in the hippocampal dentate gyrus of mice. Slides of mouse brains were washed with PBS, immersed in 0.5% Triton-X-100 for 20 min, and then blocked with 1% normal goat serum in PBS containing 0.1 % Tween 20 for 40 min at room temperature. Sections were then diluted with the primary rabbit polyclonal antibody Doublecortin (1:200, #4604, Cell Signaling Technology) and incubated overnight at 4° C. After rinsing with PBS, the specimen sections were incubated with Anti-Rabbit IgG (H+L) (1:500 A11034, Alexa Fluor 488 Conjugate) for 1 h at room temperature in the dark. Slides were washed in PBS, mounted with mounting medium with DAPI (H-1800, Vector Laboratories), and sealed with coverslips for analysis. Images were captured using a fluorescence microscope. *In vitro*, cells were discarded from the medium, washed with PBS, and fixed with 4% paraformaldehyde at room temperature. Cells were permeabilized using 0.1% Triton-X100, followed by blocking with 2% bovine serum albumin. Cells were incubated with diluted primary antibody mouse monoclonal antibody MAP-2 (1:500, sc-74421, Santa Cruz) overnight at 4° C. After washing with PBST, diluted secondary antibodies were added for 1 h and then mounted with DAPI for fluorescence microscopy.

### Statistical analysis

Data are presented as the mean ± standard error (SEM). One-way ANOVA and one-way ANOVA with repeated measures were used for the data analysis. Analysis was then performed using the Tukey, Fisher's LSD, and Scheffe tests. Student’s t-test and paired t-test were used to compare between groups. P-values were considered statistically significant at p < 0.05. All statistical analyses were performed using the SPSS version 25 software. Bar charts were made using Sigma Plot 12.0.

### Data availability statement

The original contributions presented in the study are included in the article; further inquiries can be directed to the corresponding author.

## Supplementary Material

Supplementary Figures

## References

[1] Dewan MC, Rattani A, Gupta S, Baticulon RE, Hung YC, Punchak M, Agrawal A, Adeleye AO, Shrime MG, Rubiano AM, Rosenfeld JV, Park KB. Estimating the global incidence of traumatic brain injury. J Neurosurg. 2018; 130: 1080–97. 10.3171/2017.10.JNS17352 29701556

[2] Corps KN, Roth TL, McGavern DB. Inflammation and neuroprotection in traumatic brain injury. JAMA Neurol. 2015; 72:355–62. 10.1001/jamaneurol.2014.3558 25599342 PMC5001842

[3] Kokkinou M, Kyprianou TC, Kyriakides E, Constantinidou F. A population study on the epidemiology and outcome of brain injuries in intensive care. NeuroRehabilitation. 2020; 47:143–52. 10.3233/NRE-203111 32741786

[4] Smith JA, Park S, Krause JS, Banik NL. Oxidative stress, DNA damage, and the telomeric complex as therapeutic targets in acute neurodegeneration. Neurochem Int. 2013; 62:764–75. 10.1016/j.neuint.2013.02.013 23422879 PMC3619128

[5] Davis CK, Vemuganti R. DNA damage and repair following traumatic brain injury. Neurobiol Dis. 2021; 147:105143. 10.1016/j.nbd.2020.105143 33127471

[6] Vella MA, Crandall ML, Patel MB. Acute Management of Traumatic Brain Injury. Surg Clin North Am. 2017; 97:1015–30. 10.1016/j.suc.2017.06.003 28958355 PMC5747306

[7] Nedergaard M, Takano T, Hansen AJ. Beyond the role of glutamate as a neurotransmitter. Nat Rev Neurosci. 2002; 3:748–55. 10.1038/nrn916 12209123

[8] Clements JD, Lester RA, Tong G, Jahr CE, Westbrook GL. The time course of glutamate in the synaptic cleft. Science. 1992; 258:1498–501. 10.1126/science.1359647 1359647

[9] Auger C, Attwell D. Fast removal of synaptic glutamate by postsynaptic transporters. Neuron. 2000; 28:547–58. 10.1016/s0896-6273(00)00132-x 11144363

[10] Smith JS, Fulop ZL, Levinsohn SA, Darrell RS, Stein DG. Effects of the novel NMDA receptor antagonist gacyclidine on recovery from medial frontal cortex contusion injury in rats. Neural Plast. 2000; 7:73–91. 10.1155/NP.2000.73 10709216 PMC2565364

[11] Lin CJ, Chen TH, Yang LY, Shih CM. Resveratrol protects astrocytes against traumatic brain injury through inhibiting apoptotic and autophagic cell death. Cell Death Dis. 2014; 5:e1147. 10.1038/cddis.2014.123 24675465 PMC3973229

[12] Guerriero RM, Giza CC, Rotenberg A. Glutamate and GABA imbalance following traumatic brain injury. Curr Neurol Neurosci Rep. 2015; 15:27. 10.1007/s11910-015-0545-1 25796572 PMC4640931

[13] Faden AI, Demediuk P, Panter SS, Vink R. The role of excitatory amino acids and NMDA receptors in traumatic brain injury. Science. 1989; 244:798–800. 10.1126/science.2567056 2567056

[14] Kierans AS, Kirov II, Gonen O, Haemer G, Nisenbaum E, Babb JS, Grossman RI, Lui YW. Myoinositol and glutamate complex neurometabolite abnormality after mild traumatic brain injury. Neurology. 2014; 82:521–8. 10.1212/WNL.0000000000000105 24401686 PMC3937862

[15] Salt TE, Eaton SA. Functions of ionotropic and metabotropic glutamate receptors in sensory transmission in the mammalian thalamus. Prog Neurobiol. 1996; 48:55–72. 10.1016/0301-0082(95)00047-x 8830348

[16] D’Ambrosio R, Maris DO, Grady MS, Winn HR, Janigro D. Impaired K(+) homeostasis and altered electrophysiological properties of post-traumatic hippocampal glia. J Neurosci. 1999; 19:8152–62. 10.1523/JNEUROSCI.19-18-08152.1999 10479715 PMC4066407

[17] Rothman SM. The neurotoxicity of excitatory amino acids is produced by passive chloride influx. J Neurosci. 1985; 5:1483–9. 10.1523/JNEUROSCI.05-06-01483.1985 3925091 PMC6565259

[18] Tymianski M, Charlton MP, Carlen PL, Tator CH. Source specificity of early calcium neurotoxicity in cultured embryonic spinal neurons. J Neurosci. 1993; 13:2085–104. 10.1523/JNEUROSCI.13-05-02085.1993 8097530 PMC6576557

[19] Wang CC, Wee HY, Hu CY, Chio CC, Kuo JR. The Effects of Memantine on Glutamic Receptor-Associated Nitrosative Stress in a Traumatic Brain Injury Rat Model. World Neurosurg. 2018; 112:e719–31. 10.1016/j.wneu.2018.01.140 29382619

[20] McCarty MF, Lerner A. Nutraceutical induction and mimicry of heme oxygenase activity as a strategy for controlling excitotoxicity in brain trauma and ischemic stroke: focus on oxidative stress. Expert Rev Neurother. 2021; 21:157–68. 10.1080/14737175.2021.1861940 33287596

[21] Chen Y, Swanson RA. Astrocytes and brain injury. J Cereb Blood Flow Metab. 2003; 23:137–49. 10.1097/01.WCB.0000044631.80210.3C 12571445

[22] Matute C, Domercq M, Sánchez-Gómez MV. Glutamate-mediated glial injury: mechanisms and clinical importance. Glia. 2006; 53:212–24. 10.1002/glia.20275 16206168

[23] Robel S, Buckingham SC, Boni JL, Campbell SL, Danbolt NC, Riedemann T, Sutor B, Sontheimer H. Reactive astrogliosis causes the development of spontaneous seizures. J Neurosci. 2015; 35:3330–45. 10.1523/JNEUROSCI.1574-14.2015 25716834 PMC4339349

[24] Li D, Liu N, Zhao HH, Zhang X, Kawano H, Liu L, Zhao L, Li HP. Interactions between Sirt1 and MAPKs regulate astrocyte activation induced by brain injury *in vitro* and *in vivo*. J Neuroinflammation. 2017; 14:67. 10.1186/s12974-017-0841-6 28356158 PMC5372348

[25] Zhang Z, Li D, Xu L, Li HP. Sirt1 improves functional recovery by regulating autophagy of astrocyte and neuron after brain injury. Brain Res Bull. 2019; 150:42–9. 10.1016/j.brainresbull.2019.05.005 31102754

[26] Burda JE, Bernstein AM, Sofroniew MV. Astrocyte roles in traumatic brain injury. Exp Neurol. 2016; 275:305–15. 10.1016/j.expneurol.2015.03.020 25828533 PMC4586307

[27] Chen C, Zhong X, Smith DK, Tai W, Yang J, Zou Y, Wang LL, Sun J, Qin S, Zhang CL. Astrocyte-Specific Deletion of Sox2 Promotes Functional Recovery After Traumatic Brain Injury. Cereb Cortex. 2019; 29:54–69. 10.1093/cercor/bhx303 29161339 PMC6659023

[28] Killen MJ, Giorgi-Coll S, Helmy A, Hutchinson PJ, Carpenter KL. Metabolism and inflammation: implications for traumatic brain injury therapeutics. Expert Rev Neurother. 2019; 19:227–42. 10.1080/14737175.2019.1582332 30848963

[29] Simon DW, McGeachy MJ, Bayır H, Clark RS, Loane DJ, Kochanek PM. The far-reaching scope of neuroinflammation after traumatic brain injury. Nat Rev Neurol. 2017; 13:171–91. 10.1038/nrneurol.2017.13 28186177 PMC5675525

[30] Di Pietro V, Yakoub KM, Caruso G, Lazzarino G, Signoretti S, Barbey AK, Tavazzi B, Lazzarino G, Belli A, Amorini AM. Antioxidant Therapies in Traumatic Brain Injury. Antioxidants (Basel). 2020; 9:260. 10.3390/antiox9030260 32235799 PMC7139349

[31] Pearn ML, Niesman IR, Egawa J, Sawada A, Almenar-Queralt A, Shah SB, Duckworth JL, Head BP. Pathophysiology Associated with Traumatic Brain Injury: Current Treatments and Potential Novel Therapeutics. Cell Mol Neurobiol. 2017; 37:571–85. 10.1007/s10571-016-0400-1 27383839 PMC11482200

[32] Wu TY, Lin JN, Luo ZY, Hsu CJ, Wang JS, Wu HP. 2,3,4',5-Tetrahydroxystilbene-2-O-β-D-Glucoside (THSG) Activates the Nrf2 Antioxidant Pathway and Attenuates Oxidative Stress-Induced Cell Death in Mouse Cochlear UB/OC-2 Cells. Biomolecules. 2020; 10:465. 10.3390/biom10030465 32197448 PMC7175305

[33] Ling S, Xu JW. Biological Activities of 2,3,5,4'-Tetrahydroxystilbene-2-O-β-D-Glucoside in Antiaging and Antiaging-Related Disease Treatments. Oxid Med Cell Longev. 2016; 2016:4973239. 10.1155/2016/4973239 27413420 PMC4931083

[34] Cheng J, Wang H, Zhang Z, Liang K. Stilbene glycoside protects osteoblasts against oxidative damage via Nrf2/HO-1 and NF-κB signaling pathways. Arch Med Sci. 2019; 15:196–203. 10.5114/aoms.2018.79937 30697271 PMC6348355

[35] Zhou X, Yang Q, Song F, Bi L, Yuan J, Guan S, Yang Q, Wang S. Tetrahydroxystilbene Glucoside Ameliorates Infrasound-Induced Central Nervous System (CNS) Injury by Improving Antioxidant and Anti-Inflammatory Capacity. Oxid Med Cell Longev. 2020; 2020:6576718. 10.1155/2020/6576718 31998440 PMC6977337

[36] Park SY, Jin ML, Wang Z, Park G, Choi YW. 2,3,4',5-tetrahydroxystilbene-2-O-β-d-glucoside exerts anti-inflammatory effects on lipopolysaccharide-stimulated microglia by inhibiting NF-κB and activating AMPK/Nrf2 pathways. Food Chem Toxicol. 2016; 97:159–67. 10.1016/j.fct.2016.09.010 27621050

[37] Yang XP, Liu TY, Qin XY, Yu LC. Potential protection of 2,3,5,4'-tetrahydroxystilbene-2-O-β-D-glucoside against staurosporine-induced toxicity on cultured rat hippocampus neurons. Neurosci Lett. 2014; 576:79–83. 10.1016/j.neulet.2014.05.045 24887581

[38] Fan YF, Guan SY, Luo L, Li YJ, Yang L, Zhou XX, Guo GD, Zhao MG, Yang Q, Liu G. Tetrahydroxystilbene glucoside relieves the chronic inflammatory pain by inhibiting neuronal apoptosis, microglia activation, and GluN2B overexpression in anterior cingulate cortex. Mol Pain. 2018; 14:1744806918814367. 10.1177/1744806918814367 30380983 PMC6259074

[39] Zhang X, Chen J, Graham SH, Du L, Kochanek PM, Draviam R, Guo F, Nathaniel PD, Szabó C, Watkins SC, Clark RS. Intranuclear localization of apoptosis-inducing factor (AIF) and large scale DNA fragmentation after traumatic brain injury in rats and in neuronal cultures exposed to peroxynitrite. J Neurochem. 2002; 82:181–91. 10.1046/j.1471-4159.2002.00975.x 12091479

[40] Zhang X, Chen Y, Jenkins LW, Kochanek PM, Clark RS. Bench-to-bedside review: Apoptosis/programmed cell death triggered by traumatic brain injury. Crit Care. 2005; 9:66–75. 10.1186/cc2950 15693986 PMC1065095

[41] Jiang W, Jin P, Wei W, Jiang W. Apoptosis in cerebrospinal fluid as outcome predictors in severe traumatic brain injury: An observational study. Medicine (Baltimore). 2020; 99:e20922. 10.1097/MD.0000000000020922 32590803 PMC7328954

[42] Ming GL, Song H. Adult neurogenesis in the mammalian brain: significant answers and significant questions. Neuron. 2011; 70:687–702. 10.1016/j.neuron.2011.05.001 21609825 PMC3106107

[43] La Rosa C, Parolisi R, Bonfanti L. Brain Structural Plasticity: From Adult Neurogenesis to Immature Neurons. Front Neurosci. 2020; 14:75. 10.3389/fnins.2020.00075 32116519 PMC7010851

[44] Shultz SR, McDonald SJ, Corrigan F, Semple BD, Salberg S, Zamani A, Jones NC, Mychasiuk R. Clinical Relevance of Behavior Testing in Animal Models of Traumatic Brain Injury. J Neurotrauma. 2020; 37:2381–400. 10.1089/neu.2018.6149 30907237

[45] Campolo M, Esposito E, Cuzzocrea S. A Controlled Cortical Impact Preclinical Model of Traumatic Brain Injury. Methods Mol Biol. 2018; 1727:385–91. 10.1007/978-1-4939-7571-6_30 29222798

[46] Siebold L, Obenaus A, Goyal R. Criteria to define mild, moderate, and severe traumatic brain injury in the mouse controlled cortical impact model. Exp Neurol. 2018; 310:48–57. 10.1016/j.expneurol.2018.07.004 30017882

[47] Xiong Y, Mahmood A, Chopp M. Animal models of traumatic brain injury. Nat Rev Neurosci. 2013; 14:128–42. 10.1038/nrn3407 23329160 PMC3951995

[48] Tucker LB, Velosky AG, McCabe JT. Applications of the Morris water maze in translational traumatic brain injury research. Neurosci Biobehav Rev. 2018; 88:187–200. 10.1016/j.neubiorev.2018.03.010 29545166

[49] Fox GB, Fan L, Levasseur RA, Faden AI. Sustained sensory/motor and cognitive deficits with neuronal apoptosis following controlled cortical impact brain injury in the mouse. J Neurotrauma. 1998; 15:599–614. 10.1089/neu.1998.15.599 9726259

[50] Bondi CO, Semple BD, Noble-Haeusslein LJ, Osier ND, Carlson SW, Dixon CE, Giza CC, Kline AE. Found in translation: Understanding the biology and behavior of experimental traumatic brain injury. Neurosci Biobehav Rev. 2015; 58:123–46. 10.1016/j.neubiorev.2014.12.004 25496906 PMC4465064

[51] Griesbach GS, Masel BE, Helvie RE, Ashley MJ. The Impact of Traumatic Brain Injury on Later Life: Effects on Normal Aging and Neurodegenerative Diseases. J Neurotrauma. 2018; 35:17–24. 10.1089/neu.2017.5103 28920532

[52] Chen T, Yang YJ, Li YK, Liu J, Wu PF, Wang F, Chen JG, Long LH. Chronic administration tetrahydroxystilbene glucoside promotes hippocampal memory and synaptic plasticity and activates ERKs, CaMKII and SIRT1/miR-134 *in vivo*. J Ethnopharmacol. 2016; 190:74–82. 10.1016/j.jep.2016.06.012 27275773

[53] Shen C, Sun FL, Zhang RY, Zhang L, Li YL, Zhang L, Li L. Tetrahydroxystilbene glucoside ameliorates memory and movement functions, protects synapses and inhibits α-synuclein aggregation in hippocampus and striatum in aged mice. Restor Neurol Neurosci. 2015; 33:531–41. 10.3233/RNN-150514 26409411

[54] Piot-Grosjean O, Wahl F, Gobbo O, Stutzmann JM. Assessment of sensorimotor and cognitive deficits induced by a moderate traumatic injury in the right parietal cortex of the rat. Neurobiol Dis. 2001; 8:1082–93. 10.1006/nbdi.2001.0450 11741403

[55] Stanley JL, Lincoln RJ, Brown TA, McDonald LM, Dawson GR, Reynolds DS. The mouse beam walking assay offers improved sensitivity over the mouse rotarod in determining motor coordination deficits induced by benzodiazepines. J Psychopharmacol. 2005; 19:221–7. 10.1177/0269881105051524 15888506

[56] Christian KM, Song H, Ming GL. Functions and dysfunctions of adult hippocampal neurogenesis. Annu Rev Neurosci. 2014; 37:243–62. 10.1146/annurev-neuro-071013-014134 24905596 PMC5531058

[57] Wang X, Gao X, Michalski S, Zhao S, Chen J. Traumatic Brain Injury Severity Affects Neurogenesis in Adult Mouse Hippocampus. J Neurotrauma. 2016; 33:721–33. 10.1089/neu.2015.4097 26414411 PMC4841001

[58] Tong J, Liu W, Wang X, Han X, Hyrien O, Samadani U, Smith DH, Huang JH. Inhibition of Nogo-66 receptor 1 enhances recovery of cognitive function after traumatic brain injury in mice. J Neurotrauma. 2013; 30:247–58. 10.1089/neu.2012.2493 22967270 PMC3579383

[59] Deuel TA, Liu JS, Corbo JC, Yoo SY, Rorke-Adams LB, Walsh CA. Genetic interactions between doublecortin and doublecortin-like kinase in neuronal migration and axon outgrowth. Neuron. 2006; 49:41–53. 10.1016/j.neuron.2005.10.038 16387638

[60] Chiaretti A, Barone G, Riccardi R, Antonelli A, Pezzotti P, Genovese O, Tortorolo L, Conti G. NGF, DCX, and NSE upregulation correlates with severity and outcome of head trauma in children. Neurology. 2009; 72:609–16. 10.1212/01.wnl.0000342462.51073.06 19221293

[61] Ren TT, Fan SR, Lang XY, Yu Y, Lan R, Qin XY. 2,3,5,4'-tetrahydoxystilbene-2-O-β-D-glucoside eliminates staurosporine-induced cytotoxicity by restoring BDNF-TrkB/Akt signaling axis. Int J Med Sci. 2020; 17:2207–13. 10.7150/ijms.47919 32922183 PMC7484659

[62] Lin F, Zhou Y, Shi W, Wan Y, Zhang Z, Zhang F. Tetrahydroxystilbene Glucoside Improves Neurotrophic Factors Release in Cultured Astroglia. CNS Neurol Disord Drug Targets. 2016; 15:514–9. 10.2174/1871527314666150821102025 26295829

[63] Chen X, Hu W, Lu X, Jiang B, Wang J, Zhang W, Huang C. Mechanism of 2,3,4',5-Tetrahydroxystilbene 2-O-β-D-Glucoside-Induced Upregulation of Glutamate Transporter 1 Protein Expression in Mouse Primary Astrocytes. Pharmacology. 2017; 99:153–9. 10.1159/000452672 28049198

[64] Jiang CY, Qin XY, Yuan MM, Lu GJ, Cheng Y. 2,3,5,4'-Tetrahydroxystilbene-2-O-beta-D-glucoside Reverses Stress-Induced Depression via Inflammatory and Oxidative Stress Pathways. Oxid Med Cell Longev. 2018; 2018:9501427. 10.1155/2018/9501427 30327715 PMC6169245

[65] Jarrahi A, Braun M, Ahluwalia M, Gupta RV, Wilson M, Munie S, Ahluwalia P, Vender JR, Vale FL, Dhandapani KM, Vaibhav K. Revisiting Traumatic Brain Injury: From Molecular Mechanisms to Therapeutic Interventions. Biomedicines. 2020; 8:389. 10.3390/biomedicines8100389 33003373 PMC7601301

[66] Akamatsu Y, Hanafy KA. Cell Death and Recovery in Traumatic Brain Injury. Neurotherapeutics. 2020; 17:446–56. 10.1007/s13311-020-00840-7 32056100 PMC7283441

[67] Redell JB, Maynard ME, Underwood EL, Vita SM, Dash PK, Kobori N. Traumatic brain injury and hippocampal neurogenesis: Functional implications. Exp Neurol. 2020; 331:113372. 10.1016/j.expneurol.2020.113372 32504636 PMC7803458

[68] Chang EH, Adorjan I, Mundim MV, Sun B, Dizon ML, Szele FG. Traumatic Brain Injury Activation of the Adult Subventricular Zone Neurogenic Niche. Front Neurosci. 2016; 10:332. 10.3389/fnins.2016.00332 27531972 PMC4969304

[69] Schwab N, Tator C, Hazrati LN. DNA damage as a marker of brain damage in individuals with history of concussions. Lab Invest. 2019; 99:1008–18. 10.1038/s41374-019-0199-8 30760862

[70] Lewén A, Sugawara T, Gasche Y, Fujimura M, Chan PH. Oxidative cellular damage and the reduction of APE/Ref-1 expression after experimental traumatic brain injury. Neurobiol Dis. 2001; 8:380–90. 10.1006/nbdi.2001.0396 11447995

[71] Jeggo PA, Löbrich M. DNA double-strand breaks: their cellular and clinical impact? Oncogene. 2007; 26:7717–9. 10.1038/sj.onc.1210868 18066083

[72] Li F, Wang X, Zhang Z, Zhang X, Gao P. Dexmedetomidine Attenuates Neuroinflammatory-Induced Apoptosis after Traumatic Brain Injury via Nrf2 signaling pathway. Ann Clin Transl Neurol. 2019; 6:1825–35. 10.1002/acn3.50878 31478596 PMC6764501

[73] Shao X, Yang X, Shen J, Chen S, Jiang X, Wang Q, Di Q. TNF-α-induced p53 activation induces apoptosis in neurological injury. J Cell Mol Med. 2020; 24:6796–803. 10.1111/jcmm.15333 32344470 PMC7299703

[74] Yang LY, Greig NH, Huang YN, Hsieh TH, Tweedie D, Yu QS, Hoffer BJ, Luo Y, Kao YC, Wang JY. Post-traumatic administration of the p53 inactivator pifithrin-α oxygen analogue reduces hippocampal neuronal loss and improves cognitive deficits after experimental traumatic brain injury. Neurobiol Dis. 2016; 96:216–26. 10.1016/j.nbd.2016.08.012 27553877 PMC5878046

[75] Zhang R, Sun F, Zhang L, Sun X, Li L. Tetrahydroxystilbene glucoside inhibits α-synuclein aggregation and apoptosis in A53T α-synuclein-transfected cells exposed to MPP. Can J Physiol Pharmacol. 2017; 95:750–8. 10.1139/cjpp-2016-0209 28187263

[76] Lee SY, Ahn SM, Wang Z, Choi YW, Shin HK, Choi BT. Neuroprotective effects of 2,3,5,4'-tetrahydoxystilbene-2-O-β-D-glucoside from Polygonum multiflorum against glutamate-induced oxidative toxicity in HT22 cells. J Ethnopharmacol. 2017; 195:64–70. 10.1016/j.jep.2016.12.001 27939422

[77] Zhao YY, Zhang L, Feng YL, Chen DQ, Xi ZH, Du X, Bai X, Lin RC. Pharmacokinetics of 2,3,5,4'-tetrahydroxystilbene-2-O-β-D-glucoside in rat using ultra-performance LC-quadrupole TOF-MS. J Sep Sci. 2013; 36:863–71. 10.1002/jssc.201200668 23371758

[78] Yin X, Chen C, Xu T, Li L, Zhang L. Tetrahydroxystilbene glucoside modulates amyloid precursor protein processing via activation of AKT-GSK3β pathway in cells and in APP/PS1 transgenic mice. Biochem Biophys Res Commun. 2018; 495:672–8. 10.1016/j.bbrc.2017.11.059 29129695

[79] Zhang RY, Zhang L, Zhang L, Wang YL, Li L. Anti-amyloidgenic and neurotrophic effects of tetrahydroxystilbene glucoside on a chronic mitochondrial dysfunction rat model induced by sodium azide. J Nat Med. 2018; 72:596–606. 10.1007/s11418-018-1177-y 29508255

[80] Li XX, Yu Y, Lang XY, Jiang CY, Lan R, Qin XY. 2,3,5,4'-Tetrahydroxystilbene-2-O-β-d-glucoside Restores BDNF-TrkB and FGF2-Akt Signaling Axis to Attenuate Stress-induced Depression. Neuroscience. 2020; 430:25–33. 10.1016/j.neuroscience.2020.01.025 32007553

[81] Yi CA, Wang J, Wang Y, Wu XY. Neuroprotection by 2,3,5,4'-tetrahydroxystilbene-2-O-β-D-glucoside extracts from Polygonum multiflorum against cerebral ischemia/reperfusion injury through the 5-hydroxytryptamine/5-hydroxytryptamine receptor pathway. Neuropsychiatr Dis Treat. 2019; 15:1429–38. 10.2147/NDT.S179845 31213817 PMC6549393

[82] Kajimoto M, Ledee DR, Olson AK, Isern NG, Robillard-Frayne I, Des Rosiers C, Portman MA. Selective cerebral perfusion prevents abnormalities in glutamate cycling and neuronal apoptosis in a model of infant deep hypothermic circulatory arrest and reperfusion. J Cereb Blood Flow Metab. 2016; 36:1992–2004. 10.1177/0271678X16666846 27604310 PMC5094314

[83] Zeng J, Yang GY, Ying W, Kelly M, Hirai K, James TL, Swanson RA, Litt L. Pyruvate improves recovery after PARP-1-associated energy failure induced by oxidative stress in neonatal rat cerebrocortical slices. J Cereb Blood Flow Metab. 2007; 27:304–15. 10.1038/sj.jcbfm.9600335 16736046

[84] He J, Mao J, Hou L, Jin S, Wang X, Ding Z, Jin Z, Guo H, Dai R. Minocycline attenuates neuronal apoptosis and improves motor function after traumatic brain injury in rats. Exp Anim. 2021; 70:563–9. 10.1538/expanim.21-0028 34349080 PMC8614018

[85] Miñambres E, Ballesteros MA, Mayorga M, Marin MJ, Muñoz P, Figols J, López-Hoyos M. Cerebral apoptosis in severe traumatic brain injury patients: an *in vitro*, *in vivo*, and postmortem study. J Neurotrauma. 2008; 25:581–91. 10.1089/neu.2007.0398 18363508

[86] Tsai PW, Lee YH, Chen LG, Lee CJ, Wang CC. *In Vitro* and *In Vivo* Anti-Osteoarthritis Effects of 2,3,5,4'-Tetrahydroxystilbene-2-O-β-d-Glucoside from Polygonum Multiflorum. Molecules. 2018; 23:571. 10.3390/molecules23030571 29510478 PMC6017566

[87] Luong TN, Carlisle HJ, Southwell A, Patterson PH. Assessment of motor balance and coordination in mice using the balance beam. J Vis Exp. 2011; 2376. 10.3791/2376 21445033 PMC3197288

[88] Tsenter J, Beni-Adani L, Assaf Y, Alexandrovich AG, Trembovler V, Shohami E. Dynamic changes in the recovery after traumatic brain injury in mice: effect of injury severity on T2-weighted MRI abnormalities, and motor and cognitive functions. J Neurotrauma. 2008; 25:324–33. 10.1089/neu.2007.0452 18373482

[89] Flierl MA, Stahel PF, Beauchamp KM, Morgan SJ, Smith WR, Shohami E. Mouse closed head injury model induced by a weight-drop device. Nat Protoc. 2009; 4:1328–37. 10.1038/nprot.2009.148 19713954

[90] Wu K, Huang D, Zhu C, Kasanga EA, Zhang Y, Yu E, Zhang H, Ni Z, Ye S, Zhang C, Hu J, Zhuge Q, Yang J. NT3P75-2 gene-modified bone mesenchymal stem cells improve neurological function recovery in mouse TBI model. Stem Cell Res Ther. 2019; 10:311. 10.1186/s13287-019-1428-1 31651375 PMC6814101

[91] Mosmann T. Rapid colorimetric assay for cellular growth and survival: application to proliferation and cytotoxicity assays. J Immunol Methods. 1983; 65:55–63. 10.1016/0022-1759(83)90303-4 6606682

[92] Wu J, Chien CC, Yang LY, Huang GC, Cheng MC, Lin CT, Shen SC, Chen YC. Vitamin K3-2,3-epoxide induction of apoptosis with activation of ROS-dependent ERK and JNK protein phosphorylation in human glioma cells. Chem Biol Interact. 2011; 193:3–11. 10.1016/j.cbi.2011.03.008 21453688

[93] Wu MS, Lien GS, Shen SC, Yang LY, Chen YC. N-acetyl-L-cysteine enhances fisetin-induced cytotoxicity via induction of ROS-independent apoptosis in human colonic cancer cells. Mol Carcinog. 2014 (Suppl 1); 53:E119–29. 10.1002/mc.22053 24019108

