# Supplementary material for: 2, 3, 5, 4’-tetrahydroxystilbene-2-O-beta-D-glucoside protects against neuronal cell death and traumatic brain injury-induced pathophysiology
Source: Aging (Albany NY). 2022 Mar 21;14(6):2607–27. doi: 10.18632/aging.203958 (PMC9004580; doi:10.18632/aging.203958)
Supplement: Supplementary Figures [file aging-14-203958-s001.pdf]

SUPPLEMENTARY FIGURE

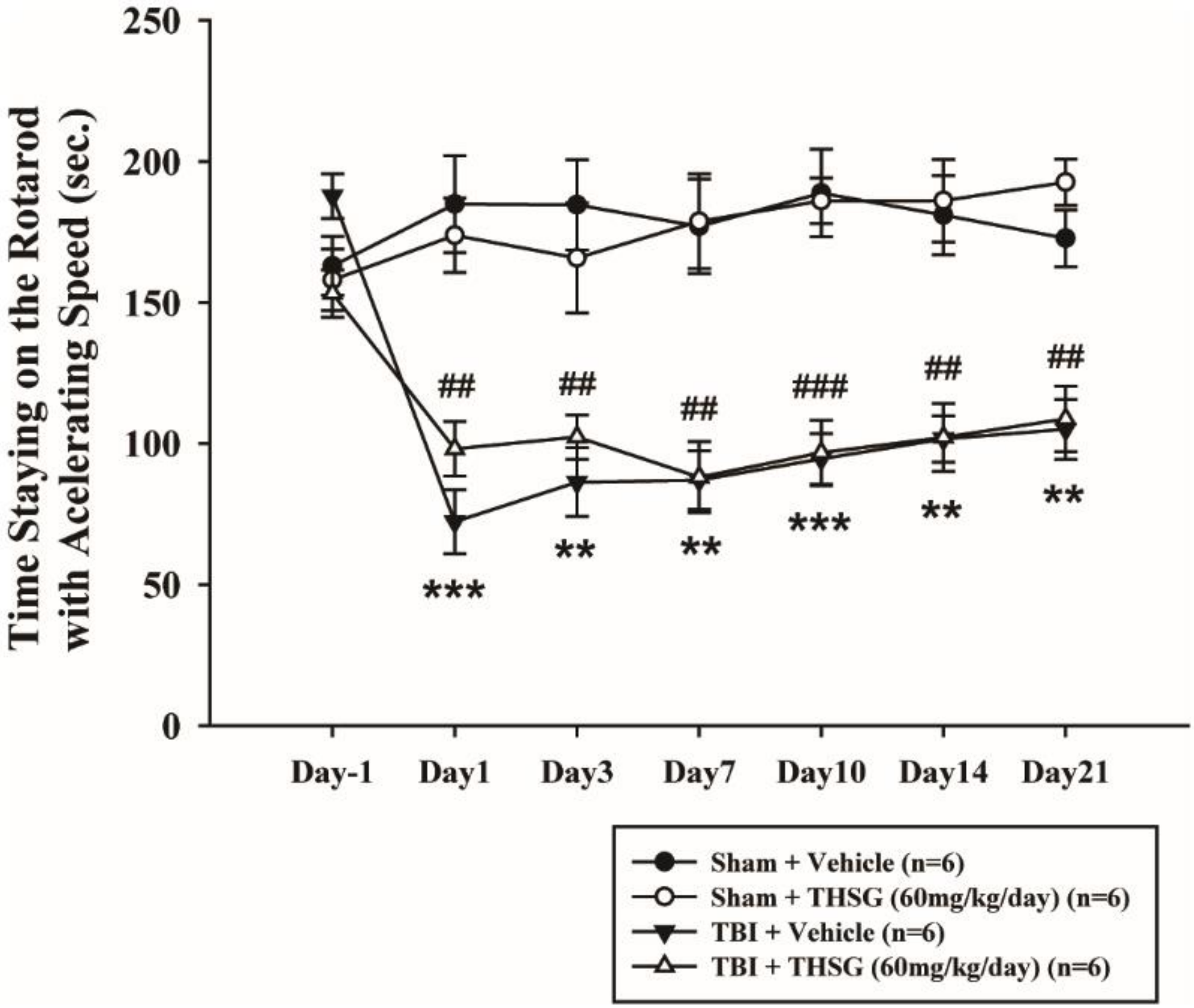

Supplementary Figure 1. Effects of THSG on rotarod performance following post-TBI. Evaluation of motor coordination by rotarod test in THSG treatments after TBI for 21 days. The time staying on rotarod with accelerating speed was measured. Data represent the mean ± SEM (n = 6 per group). **, $P < 0.01$ TBI + Vehicle vs. Sham + Vehicle group; ***, $P < 0.001$ TBI + Vehicle vs. Sham + Vehicle group; ##, $P < 0.01$ TBI + THSG vs. Sham + Vehicle group; ###, $P < 0.001$ TBI + THSG vs. Sham + Vehicle group.
